# Efficacies of anlotinib monotherapy versus gemcitabine-based chemotherapy for patients with advanced soft tissue sarcoma after the failure of anthracycline-based chemotherapy

**DOI:** 10.1007/s00432-023-05575-4

**Published:** 2024-01-31

**Authors:** Aiping Zheng, Jie Liu, Zijing Liu, Zeming Mo, Yang Fu, Yaotiao Deng, Yu Jiang

**Affiliations:** Department of Medical Oncology, Cancer Center, West China Hospital, Sichuan University, No. 37 Guo Xue Alley, Chengdu, 610041 Sichuan China

**Keywords:** Soft tissue sarcoma, Anlotinib, Gemcitabine, Chemotherapy

## Abstract

**Objective:**

The purpose of this study was to compare the antitumor efficacy of anlotinib with gemcitabine-based chemotherapy as subsequent treatment regimens in patients with advanced non-specific soft tissue sarcoma (STS) after the failure of anthracycline-based chemotherapy.

**Methods:**

Patients diagnosed with advanced STS who were treated with either anlotinib or gemcitabine-based chemotherapy between May 2009 and May 2023 in our center were eligible. All patients experienced disease progression or recurrence after the anthracycline-based chemotherapy. The primary endpoint was progression-free survival (PFS). Secondary endpoints were disease control rate (DCR), overall survival (OS) and safety.

**Results:**

We included 49 patients receiving anlotinib and 45 patients receiving gemcitabine-based chemotherapy. The median follow-up time was 76.9 weeks (range 2.9–678.9 weeks). The DCR (65.3% vs. 57.8%; *p* = 0.610), PFS (24.0 weeks vs. 18.6 weeks; *p* = 0.669) and OS (79.4 weeks vs. 87.0 weeks; *p* = 0.471) of anlotinib and gemcitabine-based chemotherapy indicated similar clinical efficacy. Moreover, exploratory subgroup analyses showed that patients with STS originating from limbs and trunk were inclined to benefit from anlotinib treatment (median PFS: 31.3 weeks vs. 12.4 weeks; *p* = 0.045). ECOG PS was an independent predictor of the PFS [Hazard Ratio (HR) 0.31; 95% confidence interval (CI) 0.11–0.85; *p* = 0.023] and OS (HR 0.26, 95%CI 0.10–0.70; *p* = 0.008) in the anlotinib group. While neutrophil-to-lymphocyte ratio (NLR) was an independent prognostic factor of the PFS (HR 0.33, 95%CI 0.11–0.98; *p* = 0.045) in the gemcitabine-based chemotherapy group. The incidence of grade 3 or higher related AEs in anlotinib and gemcitabine-based chemotherapy was 20.4% (*n* = 10) and 20.0% (*n* = 9), respectively.

**Conclusion:**

Our research suggested that anlotinib and gemcitabine-based chemotherapy showed similar clinical efficacy and safety in the subsequent treatment of advanced STS after the failure of anthracycline-based chemotherapy.

## Introduction

Soft tissue sarcomas (STS) are a large group of malignant mesenchymal tissue tumors and can occur in various parts of the body (Karakousis and Perez 1994). Although the incidence rate of soft tissue sarcoma accounts for only 1% of all solid tumors, it exhibits a wide variety of pathological types. Different types of soft tissue sarcoma have distinct clinical features, and there is also a significant variation in prognosis. This poses great challenges for clinical diagnosis and treatment (Gamboa et al. 2020). Despite advancements in treatment modalities such as surgical interventions, radiotherapy, and combination chemotherapy, over 40% of cases unfortunately experience fatal metastatic recurrence following treatment (Judson et al. 2014). For locally advanced, unresectable, and metastatic soft tissue sarcoma, chemotherapy based primarily on anthracycline drugs remains the cornerstone of first-line treatment, with reported median overall survival ranging from approximately 12–20 months (Judson et al. 2014; Ryan et al. 2016; Tap et al. 2016). For patients who have failed treatment with anthracycline-based chemotherapy, subsequent treatment is challenging. Individualized treatment approaches often depend on pathological subtypes and clinical features (Gamboa et al. 2020). Chemotherapy options may include drugs like gemcitabine, docetaxel, eribulin, trabectedin, dacarbazine and so on (Chi et al. 2018; Lin et al. 2018; Tian and Yao 2023). Alternatively, targeted therapies such as pazopanib (van Hoesel et al. 1994), regorafenib (Späth-Schwalbe et al. 2000), or tazemetostat (Liu et al. 2022) may be considered (Shimada et al. 2021). At present, there are some studies to explore whether targeted therapy combined with chemotherapy can improve clinical efficacy. According to a multicenter randomized phase 2 clinical trial, gemcitabine plus pazopanib had a better PFS (5.6 months vs. 2.0 months; *p* = 0.02) compared with pazopanib monotherapy (Nakamura et al. 2016). Due to the lack of the advantages of OS and with more severe AEs in the combination therapy group, it was not recommended to routinely apply this kind of combination regimens in advanced STS patients.

Anlotinib is a small-molecule tyrosine kinase inhibitor known for its ability to target various pathways related to tumor angiogenesis and suppress tumor growth (Zhong et al. 2023). It was approved as a second-line therapy for advanced STS patients who had failed anthracycline treatment, or as a first-line therapy for alveolar soft part sarcoma and clear cell sarcoma in China (Raungkaewmanee et al. 2012). The clinical trial (ALTER0203) showed that anlotinib had better PFS (6.3 months vs. 1.5 months; *p* < 0.001), compared to placebo (Chi et al. 2018). In addition to antiangiogenic therapy, gemcitabine-based chemotherapy is also one of the most commonly utilized treatment regimens for patients who have failed treatment with anthracycline-based chemotherapy in clinical practice. It is not clear whether anlotinib monotherapy versus gemcitabine-based chemotherapy is more effective.

In this study, we conducted a retrospective analysis to compare the antitumor efficacy of the anlotinib monotherapy and gemcitabine-based chemotherapy in STS patients who had failed anthracycline treatment. Additionally, we investigated prognostic factors that predict the efficacy of anlotinib monotherapy and gemcitabine-based chemotherapy in advanced STS patients.

## Materials and methods

### Patients

The retrospective study included patients diagnosed with STS who received anlotinib monotherapy or gemcitabine-based chemotherapy between May 2009 and May 2023 at West China Hospital, Sichuan University. The inclusion criteria were as follows: (1) pathologically confirmed locally advanced or metastatic STS; (2) experienced recurrence or disease progression after prior anthracycline-based chemotherapy. The exclusion criteria were as follows: (1) excluded the following types of sarcomas: highly chemotherapy-sensitive sarcomas such as Ewing sarcoma, non-pleomorphic rhabdomyosarcoma; chemotherapy-insensitive sarcomas such as alveolar soft part sarcoma, myxoid chondrosarcoma; specific types of sarcomas such as gastrointestinal-stromal-tumor and aggressive fibromatosis. (2) patients received other systemic anti-tumor therapies such as chemotherapy, targeted therapy and immunotherapy before or during treatment. (3) patients received radiotherapy or surgery for local lesions during treatment; (4) patients lacked detailed medical records. This study was approved by the Ethics Committee of Biomedical Research, West China Hospital of Sichuan University. The research data were obtained from the Soft Tissue Sarcoma Database of Cancer Center, West China Hospital, and the requirement for informed consent was waived.

The following demographic data of the patients were obtained: age, gender, ECOG PS (Eastern Cooperative Oncology Group Performance Status) score, location of the primary tumor, histological subtypes, pathological grade according to the French Federation of Cancer Centers Sarcoma Group (FNCLCC) systems, locally advanced and/ or metastatic stage. In addition, platelets, neutrophils, lymphocytes, and monocytes of blood samples were recorded before the first administration. Based on previous research findings, the critical thresholds have been set as follows: absolute lymphocyte count (ALC) (cells/mm^3^) at 1500 (Shimizu et al. 2020), absolute neutrophil count (ANC) (cells/mm^3^) at 4000 (Cojocaru et al. 2022), neutrophil-to-lymphocyte ratio (NLR) at 3.5 (de Juan Ferré et al. 2021), platelet-to-lymphocyte ratio (PLR) at 200 (von Mehren et al. 2020), and lymphocyte-to-monocyte ratio (LMR) at 3.0 (Smrke et al. 2020).

### Treatment

Patients received oral anlotinib once daily at an initial dose of 12 mg, adjusted to 10 mg, or 8 mg depending on adverse events (AEs), on day 1–14 in a 3-week cycle. Patients receiving gemcitabine-based chemotherapy were intravenously administered gemcitabine at a dose of 900-1000 mg/m^2^ on days 1 and 8 of a 21-day cycle (gemcitabine monotherapy), or along with intravenous administration of docetaxel at a dose of 75-100 mg/m^2^ on day 8 of a 21-day cycle (gemcitabine plus docetaxel), or along with intravenous administration of albumin-bound paclitaxel at a dose of 260 mg/m^2^ on day 8 of a 21-day cycle (gemcitabine plus albumin-bound paclitaxel). For patients who had previously received pelvic radiotherapy, gemcitabine dose was adjusted to 625 mg/m^2^. Treatment continued until disease progression or unacceptable toxicity occurred or the patient chosen to stop. Adverse events (AEs) were assessed according to the Common Terminology Criteria for Adverse Events (CTCAE), version 5.0.

### Treatment assessments

The primary endpoint was PFS. PFS was calculated from the date of drug initiation to the date of disease progression or death from any cause. Secondary endpoints were disease control rate (DCR) and overall survival (OS). DCR referred to the proportion of patients who achieve complete remission (CR), partial remission (PR), and stable disease (SD). Evaluating tumor response every two cycles based on the Response Evaluation Criteria in Solid Tumors (RECIST) version 1.1. OS was calculated from the date of drug initiation to the date of death from any cause.

### Statistical analysis

IBM SPSS Statistics 26 and GraphPad Prism 8.0 were used for statistical analysis. Baseline data and AEs data were represented using direct counting method, expressed as median (range) or frequency (percentage). PFS and OS were estimated by the Kaplan–Meier method with a 95% confidence interval (CI) and compared by Log rank test. Differences between the two groups in variables were analyzed using independent samples t-test, chi-square test, or Fisher's exact test. Univariate and multivariable analysis were conducted using the Cox proportional hazards model. Statistical significance was defined as an alpha level of 0.05 (*p* < 0.05).

## Results

### Patient characteristics and treatment

**Fig. 1:**
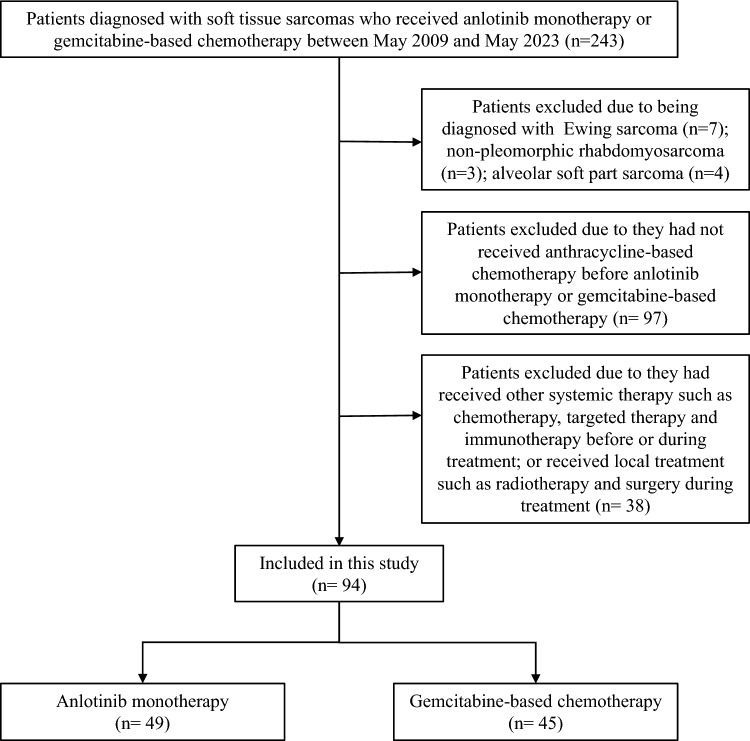
Flowchart of the study group selection process

This study included a total of 94 patients with locally advanced or metastatic STS who had experienced the failure of anthracycline-based chemotherapy (Fig. 1). Among them, 3 patients had unavailable complete blood cell counts, and medians were used as substitutes. In our study, the median follow-up time was 76.9 weeks (range 2.9–678.9 weeks). A total of 49 patients received anlotinib monotherapy, while 45 patients received gemcitabine-based chemotherapy. In the gemcitabine-based chemotherapy group, 32 patients received gemcitabine combined with docetaxel chemotherapy, 8 patients received gemcitabine combined with albumin-bound paclitaxel chemotherapy and 5 patients received gemcitabine monotherapy. The median age was 49 (range 16–76), and 58 patients were women. The ECOG PS score was mainly divided into 0–1 (*n* = 81, 86.2%) and ≥ 2 (*n* = 13, 13.8%). The primary tumor sites were the limbs and trunk (*n* = 48, 51.1%). The primary histological subtypes comprised leiomyosarcoma (*n* = 26, 27.7%), synovial sarcoma (*n* = 16, 17.0%), liposarcoma (*n* = 10, 10.6%), epithelioid sarcoma (*n* = 9, 9.6%), undifferentiated pleomorphic sarcoma (*n* = 6, 6.4%). Apart from the FNCLCC grading (*p* < 0.001), there were no significant differences in terms of age, gender, histological types, and other baseline characteristics in two groups, and the detailed information indicated in Table 1.

**Table 1:** Baseline demographics of the study patients

Characteristic	Overall (*n* = 94)	Anlotinib monotherapy (*n* = 49)	Gemcitabine-based chemotherapy (*n* = 45)	*p*-value
Median age (range)	49 (16–76)	50 (16–66)	44 (18–76)	0.627
Gender				0.921
Women	58 (61.7%)	30 (61.2%)	28 (62.2%)	
Men	36 (38.3%)	19 (38.8%)	17 (37.8%)	
ECOG PS				0.642
0–1	81 (86.2%)	43 (87.8%)	38 (84.4%)	
≥ 2	13 (13.8%)	6 (12.2%)	7 (15.6%)	
Primary lesion				0.562
Limbs and trunk	48 (51.1%)	26 (53.1%)	22 (48.9%)	
Head and neck	4 (4.3%)	2 (4.1%)	2 (4.4%)	
Abdomen and retroperitoneum	18 (19.1%)	7 (14.3%)	11 (24.4%)	
Viscera	24 (25.5%)	14 (28.6%)	10 (22.2%)	
Histology				0.091
LMS	26 (27.7%)	11 (22.4%)	15 (33.3%)	
SS^a^	16 (17.0%)	11 (22.4%)	5 (11.1%)	
LPS^b^	10 (10.6%)	2 (4.1%)	8 (17.8%)	
ES	9 (9.6%)	4 (8.2%)	5 (11.1%)	
UPS	6 (6.4%)	3 (6.1%)	3 (6.7%)	
Others^c^	27 (28.7%)	18 (36.7%)	9 (20.0%)	
FNCLCC				< 0.001
Gx, G1	46 (48.9%)	15 (30.6%)	31 (68.9%)	
G2, G3	48 (51.5%)	34 (69.4%)	14 (31.1%)	
Stage				0.135
Locally advanced	21 (22.3%)	8 (16.3%)	13 (28.9%)	
Metastatic	73 (77.7%)	41 (83.7%)	32 (71.1%)	
Number of metastatic organs				0.091
0	26 (27.2%)	9 (18.4%)	17 (37.8%)	
1	27 (28.7%)	13 (26.5%)	14 (31.1%)	
2	22 (23.4%)	14 (28.6%)	8 (17.8%)	
≥ 3	19 (20.2%)	13 (26.5%)	6 (13.3%)	
Radiotherapy history				0.873
Yes	30 (31.9%)	16 (32.7%)	14 (31.1%)	
No	64 (68.1%)	33 (67.3%)	31 (68.9%)	
Operation history				0.113
Yes	87 (92.6%)	43 (87.8%)	44 (97.8%)	
No	7 (7.4%)	6 (12.2%)	1 (2.2%)	
ALC				0.309
Low	73 (77.7%)	36 (73.5%)	37 (82.2%)	
High	21 (22.3%)	13 (26.5%)	8 (17.8%)	
ANC				0.468
Low	64 (68.1%)	35 (71.4%)	29 (64.4%)	
High	30 (31.9%)	14 (28.6%)	16 (35.6%)	
PLR				0.274
Low	51 (55.4%)	25 (51.0%)	28 (62.2%)	
High	41 (44.6%)	24 (49.0%)	17 (37.8%)	
NLR				0.889
Low	61 (64.9%)	32 (65.3%)	29 (64.4%)	
High	33 (35.1%)	17 (34.7%)	16 (35.6%)	
LMR				0.334
Low	57 (60.6%)	32 (65.3%)	25 (55.6%)	
High	37 (39.4%)	17 (34.7%)	20 (44.4%)	

*ECOG PS* Eastern cooperative oncology group performance status; *FNCLCC* French Federation of cancer centers sarcoma group; *ALC* absolute lymphocyte count; *ANC* absolute neutrophil count; *NLR* neutrophil-to-lymphocyte ratio; *PLR* platelet-to-lymphocyte ratio; *LMR* lymphocyte-to-monocyte ratio

^a^Includes synovial sarcoma, spindle cell synovial sarcoma

^b^Includes dedifferentiated liposarcoma, myxoid liposarcoma, myxoid liposarcoma, well-differentiated liposarcoma

^c^Includes myxofibrosarcoma, fibrosarcoma, sclerosing epithelioid fibrosarcoma, malignant phyllodes tumor, undifferentiated sarcoma, spindle cell undifferentiated sarcoma, spindle cell sarcoma, malignant peripheral nerve sheath tumour, malignant solitary fibrous tumor, endometrial stromal sarcoma, pleomorphic rhabdomyosarcoma, spindle cell/sclerosing rhabdomyosarcoma, epithelioid haemangioendothelioma, myoepithelial carcinoma

### Efficacy

In the anlotinib group, 1 patient (2.0%) achieved CR, 1 patient (2.0%) achieved PR, 30 patients (61.3%) achieved SD, and 17 patients (34.7%) experienced progressive disease (PD), yielding a DCR of 65.3%. In the gemcitabine-based chemotherapy group, 2 patients (4.4%) achieved PR, 24 patients (53.3%) achieved SD, and 19 patients (42.2%) experienced PD, yielding a DCR of 57.8%. The anlotinib and gemcitabine-based chemotherapy groups did not differ significantly in DCR (*p* = 0.610, Table 2).

**Table 2:** Treatment response of the two groups

	Anlotinib monotherapy (*n* = 49)	Gemcitabine-based chemotherapy (*n* = 45)	*p*-value
DCR	32 (65.3%)	26 (57.8%)	0.610
CR	1 (2.0%)	0	
PR	1 (2.0%)	2 (4.4%)	
SD	30 (61.3%)	24 (53.3%)	
PD	17 (34.7%)	19 (42.2%)	

*DCR* disease control rate; *CR* complete remission; *PR* partial remission; *SD* stable disease; *PD* progressive disease

**Fig. 2:**
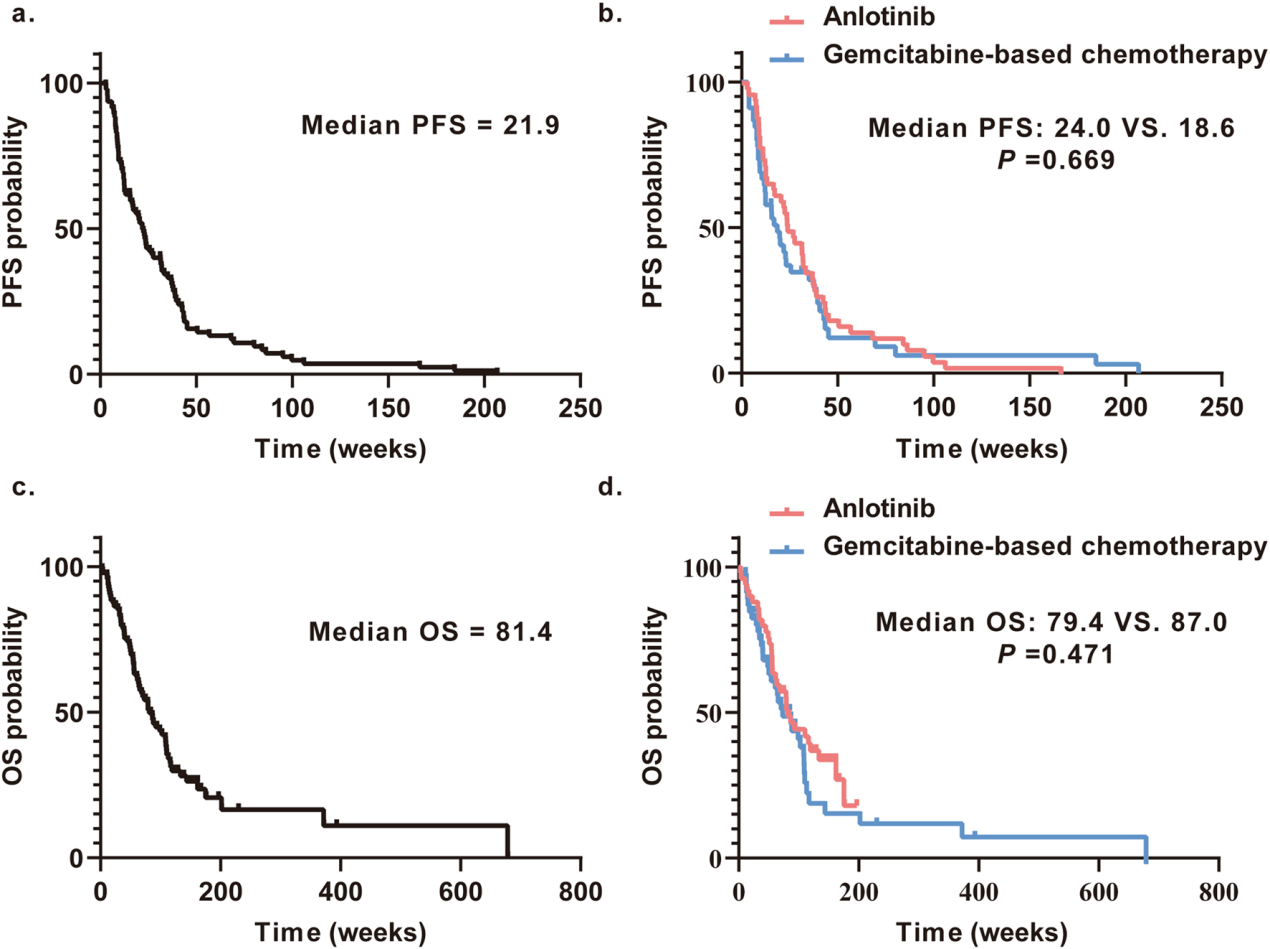
OS and PFS for advanced STSs patients. **a** PFS for all STSs patients; **b** Comparison of PFS among the anlotinib monotherapy and gemcitabine-based chemotherapy groups. There were no differences among the two groups; **c** OS for all STSs patients; **d** Comparison of OS among the anlotinib monotherapy and gemcitabine-based chemotherapy groups. There were no differences among the two groups. STS, soft tissue sarcoma; PFS, progression-free survival; OS, overall survival

All patients had a median PFS of 21.9 weeks (95% CI 15.87–27.85; Fig. 2A). The median PFS for anlotinib was 24.0 weeks (95%CI 17.54–30.46), and for gemcitabine-based chemotherapy was 18.6 weeks (95%CI 12.76–24.38). There was no significant difference in median PFS (HR 0.91; 95% CI 0.60–1.39; *p* = 0.669; Fig. 2B). All patients had a median OS of 81.4 weeks (95% CI 60.72–102.14; Fig. 2C). The median OS for anlotinib was 79.4 weeks (95%CI 54.47–104.38), and for gemcitabine-based chemotherapy was 87.0 weeks (95%CI 46.98–127.02). There was no significant difference in median OS for the selection of drugs (HR 0.83; 95% CI 0.51–1.36; *p* = 0.471; Fig. 2D).

### Subgroup analysis and exploratory analysis

As shown in Tables 3 and 4, in anlotinib group, univariate and multivariable analysis indicated that ECOG PS was an independent predictor of the PFS (HR 0.31; 95%CI 0.11–0.85; *p* = 0.023) and OS (HR 0.26, 95%CI 0.10–0.70; *p* = 0.008). Moreover, ANC was an independent predictor of the OS (HR 0.44, 95%CI 0.20–0.96; *p* = 0.039) in anlotinib group. While in the gemcitabine-based chemotherapy group, univariate and multivariable analysis indicated that NLR was an independent prognostic factor for PFS (HR 0.33; 95%CI 0.11–0.98; *p* = 0.045).

**Table 3:** Univariate and multivariate analyses for PFS in anlotinib monotherapy and gemcitabine-based chemotherapy

Characteristic	Anlotinib monotherapy (*n* = 49)				Gemcitabine-based chemotherapy (*n* = 45)			
	Univariate analyses		Multivariate analyses		Univariate analyses		Multivariate analyses	
	HR (95%CI)	*p*-value	HR (95%CI)	*p*-value	HR (95%CI)	*p*-value	HR (95%CI)	*p*-value
Gender								
Women (vs men)	1.38 (0.77–2.50)	0.284			1.24 (0.65–2.36)	0.516		
ECOG PS								
0–1 (vs ≥ 2)	0.28 (0.09–0.57)	**0.001**	0.31 (0.11–0.85)	**0.023**	0.82 (0.34–1.97)	0.654		
Primary lesion								
Limbs and trunk (vs others)	0.89 (0.50–1.60)	0.699			0.53 (0.27–1.04)	0.066	0.59 (0.28–1.21)	0.111
Histology								
LMS/SS (vs others)	0.88 (0.49–1.57)	0.663			0.93 (0.50–1.75)	0.825		
FNCLCC								
Gx, G1 (vs G2, G3)	0.85 (0.46–1.59)	0.617			0.87 (0.44–1.73)	0.699		
Radiotherapy history								
Yes (vs no)	0.98 (0.54–1.80)	0.950			0.75 (0.39–1.46)	0.399		
Operation history								
Yes (vs no)	0.92 (0.36–2.34)	0.853			–	0.309		
Stage								
Locally advanced (vs others)	0.56 (0.23–1.32)	0.184			0.84 (0.42–1.71)	0.639		
ALC								
Low (vs high)	0.84 (0.43–1.62)	0.596			0.68 (0.30–1.57)	0.369		
ANC								
Low (vs high)	0.45 (0.23–0.87)	**0.018**	0.59 (0.28–1.23)	0.158	0.52 (0.24–1.09)	0.081	0.49 (0.13–1.79)	0.281
PLR								
Low (vs high)	0.97 (0.55–1.73)	0.922			0.85 (0.44–1.65)	0.628		
NLR								
Low (vs high)	0.96 (0.52–1.75)	0.882			0.41 (0.21–0.83)	**0.013**	0.33 (0.11–0.98)	**0.045**
LMR								
Low (vs high)	1.47 (0.79–2.73)	0.225			1.05 (0.55–2.00)	0.878		

Bold indicates that the *p* value is less than 0.05, which is statistically significant

*PFS* progression-free survival; *ECOG PS* Eastern cooperative oncology group performance status; *LMS* leiomyosarcoma; *FNCLCC* French Federation of cancer centers sarcoma group; *ALC* absolute lymphocyte count; *ANC* absolute neutrophil count; *NLR* neutrophil-to-lymphocyte ratio; *PLR* platelet-to-lymphocyte ratio; *LMR* lymphocyte-to-monocyte ratio;

**Table 4:** Univariate and multivariate analyses for OS in anlotinib monotherapy and gemcitabine-based chemotherapy

Characteristic	Anlotinib monotherapy (*n* = 49)				Gemcitabine-based chemotherapy (*n* = 45)			
	Univariate analyses		Multivariate analyses		Univariate analyses		Multivariate analyses	
	HR (95%CI)	*p*-value	HR (95%CI)	*p*-value	HR (95%CI)	*p*-value	HR (95%CI)	*p*-value
Gender								
Women (vs men)	1.17 (0.58–2.34)	0.659			1.38 (0.69–2.75)	0.363		
ECOG PS								
0–1 (vs ≥ 2)	0.22 (0.09–0.56)	**0.001**	0.26 (0.10–0.70)	**0.008**	0.91 (0.39–2.10)	0.817		
Primary lesion								
Limbs and trunk (vs others)	0.74 (0.37–1.50)	0.410			0.74 (0.38–1.46)	0.384		
Histology								
LMS/SS (vs others)	0.93 (0.47–1.84)	0.830			1.00 (0.49–2.03)	1.00		
FNCLCC								
Gx, G1 (vs G2, G3)	0.69 (0.32–1.49)	0.343			0.99 (0.47–2.07)	0.972		
Radiotherapy history								
Yes (vs no)	0.75 (0.37–1.53)	0.433			0.67 (0.32–1.41)	0.286		
Operation history								
Yes (vs no)	0.91 (0.32–2.60)	0.859			–	0.582		
Stage								
Locally advanced (vs others)	0.99 (0.35–2.84)	0.985			0.66 (0.31–1.43)	0.295		
ALC								
Low (vs high)	0.52 (0.25–1.07)	0.076	0.63 (0.28–1.40)	0.256	0.65 (0.28–1.53)	0.328		
ANC								
Low (vs high)	0.50 (0.24–1.06)	0.072	0.44 (0.20–0.96)	**0.039**	0.37 (0.18–0.78)	**0.008**	0.41 (0.14–1.19)	0.102
PLR								
Low (vs high)	0.95 (0.48–1.92)	0.892			0.66 (0.33–1.32)	0.239		
NLR								
Low (vs high)	0.96 (0.47–1.95)	0.904			0.50 (0.25–1.02)	0.056	0.88 (0.32–2.44)	0.798
LMR								
Low (vs high)	1.73 (0.80–3.76)	0.165			1.12 (0.57–2.21)	0.751		

Bold indicates that the *p* value is less than 0.05, which is statistically significant

*OS* overall survival; *ECOG PS* Eastern cooperative oncology group performance status; *LMS* leiomyosarcoma; *FNCLCC* French Federation of cancer centers sarcoma group; *ALC* absolute lymphocyte count; *ANC* absolute neutrophil count; *NLR* neutrophil-to-lymphocyte ratio; *PLR* platelet-to-lymphocyte ratio; *LMR* lymphocyte-to-monocyte ratio

**Fig. 3:**
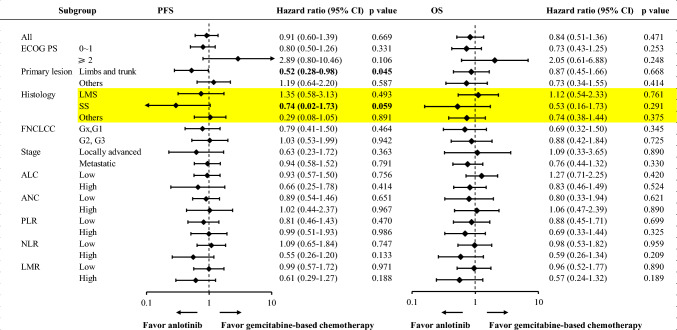
Outcomes of subgroups of soft tissue sarcoma patients. Progression-free survival (PFS) and overall survival (OS) in patient subgroups are defined by key baseline characteristics. PFS, progression-free survival; OS, overall survival; ECOG PS, Eastern Cooperative Oncology Group Performance Status; LMS, leiomyosarcoma; SS, synovial sarcoma; FNCLCC, French Federation of Cancer Centers Sarcoma Group; ALC, absolute lymphocyte count; ANC, absolute neutrophil count; NLR, neutrophil-to-lymphocyte ratio; PLR, platelet-to-lymphocyte ratio; LMR, lymphocyte-to-monocyte ratio

Additionally, we conducted further exploratory subgroup analyses to explore which subgroups were inclined to benefit from anlotinib monotherapy or gemcitabine-based chemotherapy. As shown in Fig. 3, STS originating in the extremities and trunk were more likely to benefit from anlotinib monotherapy. Moreover, we observed longer PFS with anlotinib monotherapy among synovial sarcoma, although the difference was marginally significant.

### Crossover treatment

After the disease progression, 10 patients (20.4%) in the anlotinib group received subsequent treatment with gemcitabine/docetaxel, the median PFS and OS of subsequent treatment were 33.6 weeks and 96.1 weeks respectively. 9 patients (20.0%) in the gemcitabine-based chemotherapy group received subsequent treatment with anlotinib, the median PFS and OS of subsequent treatment were 21.0 weeks and 63.0 weeks respectively. There was no significant difference in PFS (*p* = 0.928) and OS (*p* = 0.773) between the two crossover treatment groups.

### Treatment-related toxicities

In the anlotinib monotherapy group, the incidence of grade 3 or higher related AEs was 20.4% (*n* = 10), primarily consisting of hypertension (*n* = 5, 10.2%), pneumothorax (*n* = 3, 6.1%), weight loss (*n* = 2, 4.1%) and neutropenia (*n* = 1, 2.0%). In the gemcitabine-based chemotherapy group, the incidence of grade 3 or higher related AEs was 20.0% (*n* = 9), primarily involving hematologic AEs such as thrombocytopenia (*n* = 2, 4.4%), neutropenia (*n* = 3, 6.7%), leukopenia (*n* = 3, 6.7%), anemia (*n* = 2, 4.4%), rash (*n* = 2, 4.4%) and diarrhea (*n* = 1, 2.2%).

## Discussions

In this study, we compared the antitumor efficacy of anlotinib and gemcitabine-based chemotherapy in advanced STS patients who experienced the failure of anthracycline-based chemotherapy. The results showed that anlotinib monotherapy and gemcitabine-based chemotherapy indicated similar DCR (65.3% vs. 57.8%; *p* = 0.610), PFS (24.0 weeks vs. 18.6 weeks; *p* = 0.669) and OS (79.4 weeks vs. 87.0 weeks; *p* = 0.471).

At present, antiangiogenic therapy has become a crucial treatment option for advanced STS patients who experienced failure of anthracycline-based chemotherapy. According to some randomized clinical trials, pazopanib (PALETTE) (van der Graaf et al. 2012), regorafenib (REGOSARC) (Mir et al. 2016) and anlotinib (ALTER0203) (Chi et al. 2018) all exhibited anti-tumor efficacy, contributed to improving PFS (pazopanib: 4.6 months vs. 1.6 months, *p* < 0.001; regorafenib: 3.7 months vs. 1.8 months, *p* < 0.001; anlotinib: 6.3 months vs. 1.5 months; *p* < 0.001). However, these clinical studies used placebo as the control group. In the study, we chose gemcitabine-based chemotherapy as the control group, the results showed anlotinib monotherapy and gemcitabine-based chemotherapy had similar PFS and OS. This implies that in the era of antiangiogenic therapy for advanced STS, we still cannot abandon posterior line chemotherapy.

Chemotherapy based on gemcitabine were first reported for the treatment of advanced STS in 2002. Quite interestingly, the combination of gemcitabine followed by docetaxel had a synergistic effect, while docetaxel followed by gemcitabine had an antagonistic effect (Hensley et al. 2002). The combination of gemcitabine and docetaxel resulted in longer PFS (6.2 months vs. 3.0 months) and OS (17.9 months vs. 11.5 months) compared to the use of gemcitabine alone (Maki et al. 2007). A recent retrospective study compared the differences in the clinical efficacy of pazopanib versus gemcitabine plus docetaxel as second-line treatments for advanced STS, the results showed pazopanib monotherapy or gemcitabine plus docetaxel could achieve similar PFS (4.5 months vs. 3.0 months; *p* = 0.593) and OS (12.6 months vs. 14.2 months; *p* = 0.362) (Kim et al. 2019). Similar with these results, our study indicated that the anlotinib group achieved similar PFS (24.0 weeks vs. 18.6 weeks; *p* = 0.669) and OS (79.4 weeks vs. 87.0 weeks; *p* = 0.471) compared to the gemcitabine-based chemotherapy group. These results suggested that antiangiogenic drug monotherapy or gemcitabine-based chemotherapy could achieve comparable efficacy in second-line therapy following anthracycline treatment failure.

Some researchers further explored the clinical efficacy of chemotherapy in combination with antiangiogenic therapy for advanced STS. In previous phase 2 randomized clinical trials, gemcitabine plus pazopanib showed a better PFS compared to pazopanib monotherapy (5.6 months vs. 2.0 months, *p* = 0.02) or gemcitabine (4.5 months vs. 1.6 months; *p* = 0.017) (Ryan et al. 2020; Schmoll et al. 2021). According to these studies, the PFS of gemcitabine combined with antiangiogenic therapy seems to be superior to gemcitabine monotherapy or pazopanib monotherapy, but the OS did not show a significant improvement. Additionally, the combination therapy led to a higher rate of AEs. Recently, a retrospective study found that the PFS (6.8 months vs. 5.8 months; *p* = 0.39) and OS (13.3 months vs. 14.7 months; *p* = 0.75) of gemcitabine plus anlotinib were similar to gemcitabine plus docetaxel (Liu et al. 2022). However, the gemcitabine plus anlotinib group had a significantly higher incidence of grade 3 AEs compared to the gemcitabine plus docetaxel group. The above studies may suggest that in second-line therapy following anthracycline treatment failure, the combination of gemcitabine with anti-angiogenic drugs could achieve efficacy similar to gemcitabine plus other chemotherapeutic agents, but could be superior to either antiangiogenic monotherapy or gemcitabine monotherapy. The value of anlotinib combined with chemotherapy in the treatment of advanced STS needs to be clarified through large-scale prospective studies.

Due to the heterogeneity of STS, distinct treatment regimens may exhibit variations in efficacy across different pathological subtypes. In the ALTER0203 study, anlotinib showed significant benefits for specific pathological subtypes, including leiomyosarcoma (PFS: 5.8 months) and synovial sarcoma (PFS: 5.7 months) (Chi et al. 2018). Similar to the ALTER0203, our research findings indicated that leiomyosarcoma (PFS: 26.86 weeks) and synovial sarcoma (PFS: 38.00 weeks) exhibited better PFS following treatment with anlotinib monotherapy. A retrospective study indicated that there was no significant difference in PFS and OS among patients with leiomyosarcomas between gemcitabine plus docetaxel and pazopanib (Kim et al. 2019). In our study, we also observed similar PFS (26.86 weeks vs. 23.14 weeks; *p* = 0.491) and OS (63.29 weeks vs. 60.71 weeks; *p* = 0.283) for leiomyosarcomas between the two groups. Results from a phase 3 study (APROMISS) evaluating the clinical efficacy of anlotinib compared to dacarbazine in patients with metastatic or recurrent advanced synovial sarcoma were presented at the American Society of Clinical Oncology (ASCO) 2021 Annual Meeting (Van Tine et al. 2021). The study revealed that the PFS (2.89 months vs. 1.64 months; *p* = 0.002) for the anlotinib treatment group was significantly better than the dacarbazine treatment group. Similar to these results, our study found that in patients with synovial sarcoma, the anlotinib group showed a better PFS (38.0 weeks vs. 8.7 weeks; *p* = 0.059) compared to the gemcitabine-based chemotherapy group. Therefore, anlotinib may be a reasonable second-line treatment option for synovial sarcoma.

Despite similar survival times, we need to explore individualized treatment. gemcitabine plus docetaxel had a higher objective response rate than pazopanib (Kim et al. 2019). This suggested that gemcitabine plus docetaxel may be considered for patients who needed to reduce tumor burden and improve symptoms as soon as possible. However, our study found that anlotinib monotherapy and gemcitabine-based chemotherapy had similar objective response rate, which may be related to insufficient sample size. In addition, our exploratory subgroup analyses found that STS originating in the extremities and trunk were more likely to benefit from anlotinib monotherapy. In our study, the predominant pathological subtypes of sarcomas originating in the extremities and trunk were primarily leiomyosarcoma and synovial sarcoma. According to ALTER0203, leiomyosarcoma and synovial sarcoma were more sensitive to anlotinib treatment (Chi et al. 2018), and our results also showed that synovial sarcoma with anlotinib monotherapy was associated with longer PFS (38.0 weeks vs. 8.7 weeks; *p* = 0.059) compared with gemcitabine-based chemotherapy. In clinical practice, clinicians may also need to consider AEs and patient preferences to select gemcitabine-based chemotherapy or antiangiogenic therapy. Our study showed that the main AEs of anlotinib was hypertension, while the main AEs of chemotherapy was hematological toxicities. Thus, elderly patients with normal blood pressure may be more inclined to choose anlotinib treatment.

The ECOG performance status is an important indicator in cancer care. It relies on an evaluation of a patient's everyday functional capabilities and is frequently employed to forecast whether a patient can endure and positively respond to subsequent treatment. A poor ECOG performance status is often related to worse clinical outcomes in various cancers. A retrospective clinical study revealed that in advanced STS patients receiving anlotinib treatment, those with a worse ECOG performance status (ECOG PS ≥ 2) had poorer survival outcomes (median PFS: 3.1 months vs. 2.0 months, *p* = 0.161; median OS: 7.1 months vs. 4.1 months, *p* = 0.234) (Zhang et al. 2022). Similar findings were also observed in non-small cell lung cancer patients (Wu et al. 2019). Consistently, our findings demonstrated that a favorable ECOG performance status (ECOG PS) is an independent predictive factor for better PFS (26.86 weeks vs. 7.86 weeks, *p* = 0.001) and OS (86.57 weeks vs. 13.43 weeks, *p* = 0.026) of STS patients receiving anlotinib. However, in the gemcitabine-based chemotherapy group, there were no significant differences in PFS and OS for ECOG performance status. According to the report, for patients with ECOG PS = 0 in leiomyosarcoma, gemcitabine/docetaxel showed better response and survival (Bay et al. 2006). In our study, patients with ECOG PS = 0 and ECOG PS = 1 were grouped together into the database. We were unable to conduct a more detailed analysis of the prognosis for patients with an ECOG = 0. This might be why ECOG PS was not a prognostic factor in the gemcitabine-based chemotherapy group in our study.

NLR is a marker of systemic inflammation and reflects the anti-tumor immune status (Rosenberg 2001). A high NLR value is generally associated with poor prognosis in various types of cancer (Guthrie et al. 2013; Templeton et al. 2014; Baert et al. 2018). In recent years, there has been a growing number of studies examining the correlation between NLR and the prognosis of STS (Cheng et al. 2019). It has been reported that preoperative low NLR may predict better clinical out comes after resection of STS (Liang et al. 2018; Teck Seo et al. 2019), and low NLR may be an independent predictor of durable clinical benefit and better OS of eribulin and pazopanib for STS (Sato et al. 2021). Furthermore, a study found that eribulin or trabectedin for patients with low NLR and eribulin for patients with low PLR were associated with longer OS (Shimada et al. 2021). Our results are consistent with these findings. In the study, we also found low pre-treatment NLR may be an independent predictive marker for better PFS (20.00 weeks vs. 12.14 weeks, *p* = 0.031) and OS (108.43 weeks vs. 60.71 weeks, *p* = 0.009) in patients receiving gemcitabine/docetaxel treatment for STS.

Our study has some limitations. First of all, the sample size was not large. Secondly, this study is a retrospective study from a single institution, which may introduce selection bias in the subjects, potentially influencing the results. In particular, some selection bias can’t be avoided. For example, the selection of drugs, including dosage and duration of treatment, depends on the physician's professional judgment and the patient's preferences. After experiencing the failure of anthracycline-based chemotherapy, some patients received gemcitabine monotherapy, while some received gemcitabine plus other chemotherapeutic agents. Due to the retrospective nature of this study, quality of life data was unavailable for many patients. Thirdly, anlotinib was used in our hospital since 2018, whereas the earliest gemcitabine-based chemotherapy group we enrolled was in 2009. Such lengthy time intervals may affect the results. However, we also analyzed the efficacy of cross-treatment between the two groups, and the results showed no difference. Moreover, some patients in gemcitabine-based chemotherapy group did not undergo assessment for FNCLCC grading. This led to differences between the two groups in terms of this baseline characteristic. To sum up, our research findings call for further validation through large-scale matched retrospective cohort study or multicenter prospective studies.

## Conclusion

In summary, our research suggested that anlotinib and gemcitabine-based chemotherapy showed similar clinical efficacy in patients with advanced STS who experienced disease progression or metastasis after the anthracycline-based chemotherapy. Moreover, STS originating in the extremities and trunk were inclined to benefit from anlotinib monotherapy. These findings should be validated through further studies, especially large-scale multicenter retrospective research and prospective study.

## Data Availability

All data are available from the corresponding author on reasonable request.
